# A novel scoring system for prognostic prediction in d-galactosamine/lipopolysaccharide-induced fulminant hepatic failure BALB/c mice

**DOI:** 10.1186/1471-230X-9-99

**Published:** 2009-12-30

**Authors:** Bo Feng, Sheng Ming Wu, Sa Lv, Feng Liu, Hong Song Chen, Yan Gao, Fang Ting Dong, Lai Wei

**Affiliations:** 1 Hepatology Institute, Peking University People's Hospital, No.11 Xizhimen South Street, Beijing 100044, PR China; 2 National Center of Biomedical Analysis, No.27 Taiping Road, Beijing 100039, PR China

## Abstract

**Background:**

It is frequently important to identify the prognosis of fulminant hepatic failure (FHF) patients as this will influence patient management and candidacy for liver transplantation. Therefore, a novel scoring system based on metabonomics combining with multivariate logistic regression was developed to predict the prognosis of FHF mouse model.

**Methods:**

BALB/c mice were used to construct FHF model. Parts of plasma were collected at 4, 5, and 6-h time points after treatment, respectively, and detected using gas chromatography/time-of-flight mass spectrometry (GC/TOFMS). The acquired data were processed using partial least square discriminant analysis (PLS-DA). The metabolic markers identified were used to construct a scoring system by multivariate regression analysis.

**Results:**

28 mice of survival group and 28 of dead group were randomly selected and analyzed. PLS regression analysis showed that both the PLS models of 5 h and 6 h after d-galactosamine/lipopolysaccharide treatment demonstrated good performances. Loadings plot suggested that phosphate, beta-hydroxybutyrate (HB), urea, glucose and lactate concentrations in plasma had the highest weightings on the clustering differences at the three time points. By the multivariate logistic regression analysis, the death/survival index (DSI) was constructed based on relative concentrations of HB, urea and phosphate. It provided general accurate rate of prediction of 93.3% in the independent samples.

**Conclusions:**

The novel scoring system based on metabonomics combining with multivariate logistic regression is accurate in predicting the prognosis of FHF mouse model and may be referred in clinical practice as a more useful prognostic tool with other available information.

## Background

Fulminant hepatic failure (FHF) is a syndrome characterized by an abrupt onset, hepatic encephalopathy and coagulation abnormality in the absence of preexistent liver disease [1]. The syndrome carries high morbidity and mortality. Orthotopic liver transplantation (OLT) has been increasingly the most important way for FHF, which decreased fatality rate with FHF from 85% to 35% [2]. Unfortunately, the application of OLT has remained low for its higher expense and less donor organs, and a high percentage of patients die before an organ is available [3]. Alternatively, with the earlier use of artificial liver supporting system (ALSS), the nonsurgical medicinal therapy was increasingly improved. Early diagnosis and prognosis identification of FHF patients is likely to result in an intervention at an earlier stage of the disease and, thus, an increased likelihood of treatment success.

In previous studies, individual compounds including lactate, phosphorus among others were proposed to predict patient outcome in FHF [4,5]. However, their prognostic value is debatable [6]. Application of multivariate models has been improved prediction accuracy of FHF outcome. The Clichy criteria developed in patients with hepatitis B consisted of factor V level, patient age, HBsAg and serum alfa-fetoprotein level. The King's College Hospital (KCH) criteria, which was performed based on a retrospective analysis of 585 FHF patients, have been the most widely applied [7]. However, a retrospective study about both criteria in FHF patients reported lower predictive values with the positive predictive values (PPV) at 73% to 79% and the negative predictive values (NPV) at < 50%[4]. The Model for End-Stage Liver Disease (MELD) scoring system is a reliable measure of short-term mortality risk in patients with end-stage chronic liver disease. However, as a predictor of death from FHF, MELD score did not provide more information than the King's College Hospital criteria or international normalized ratio (INR) alone [8]. Therefore, better prognostic model to identify FHF patients who are likely to survive is needed.

After proposed by Nicholson in 1999, metabonomics has increasingly attained attention in recent years, applied to study various human diseases such as coronary artery disease and cancers, and provided new insights into the regulatory investigations [9-11]. One of the most important objectives of metabonomic studies is to identify novel metabolic biomarkers from biofluids in various physiological and pathological conditions. In the previous studies, we reported the dynamics of metabolic profiles in d-galactosamine/lipopolysaccharide (GalN/LPS)-treated BALB/c mouse model, and identified 5 biomarkers including phosphate, β-hydroxybutyrate (HB), urea, glucose and lactate which may constitute a set of markers for the early diagnosis and prognosis of FHF [12,13]. But how to predict the prognosis based on plasma levels of these metabolic biomarkers? In this study, using metabonomic methods based on gas chromatography/time-of-flight mass spectrometry (GC/TOFMS) combined with multivariate logistic regression analysis, we aimed to derive a regression model consisted of several metabolic biomarkers for monitoring FHF, and make a basis of related investigations in clinical practice.

## Methods

### Materials

Male BALB/c mice (18-22 g) were purchased from the Academy of Military Medical Sciences (Beijing, China), housed in a standard animal laboratory with constant conditions of temperature, humidity, and 12 h light-dark cycles, and allowed free access to standard laboratory chow and water. Animal studies were carried out in accordance with the Chinese National Research Council guidelines and approved by the Subcommittee on Research Animal Care and Laboratory Animal Resources of the Peking University People's Hospital. Absolute ethanol and chloroform were obtained from Sigma (Sigma-Aldrich Chemical Co. Steinheim, Germany) and used in the extraction of polar metabolites. *N*-methyl-*N-*trimethylsilyltrifluoroacetamide (MSTFA), methoxyamine hydrochloride and pyridine were obtained from Sigma and used to derivatize pure metabolites. Ribitol was used as an internal reference. Doubly distilled water was used in the preparation of standard and sample solutions.

### Construction of experimental FHF model

The FHF model was established as described previously [13]. The model group was intraperitoneally treated with 0.6 g/kg GalN followed by 8 μg/kg LPS, and GalN/LPS treated mice were classed into a survival group and a dead group. Five mice of each group were randomly selected for collection of retro-orbital blood at 4, 5 and 6 h after treatment. Additional samples obtained at 5 h after treatment from 23 mice of the survival group and 23 mice of the dead group were also analyzed. Blood was collected into tubes containing 10 μL solution of 4% EDTA, immediately placed on ice and centrifuged at 3000 rpm for 10 min at 4°C. Plasma samples were stored at -80°C until metabonomic analysis.

### Assessment of liver injury

Plasma levels of alanine aminotransferase (ALT), aspartate aminotransferase (AST) and T-bilirubin (TBIL), measured with a 7170A automatic analyzer (Hitachi, Japan), were used to assess the extent of liver injury.

### Plasma samples preprocessing and GC/TOFMS analysis

In this study, processing of plasma samples followed a protocol described in ref. [13]. Briefly, the thawed plasma samples were quenched by pure methanol and extracted by chloroform. The upper phase was separated and then evaporated to dryness under a stream of N2 gas. At last, the precipitation was derivatized by methoximation and trimethylsilylation.

The GC/TOFMS system consists of an HP 6890 gas chromatograph and a time-of-flight mass spectrometer (Waters Co., Milford, Massachusetts, USA). As described in previous study [13], the pretreated plasma samples were analyzed. Identity of GC/MS detected peaks was established by comparing their mass spectra and retention index with those available in the NIST02 library and those of commercially available reference compounds. As for some overlapping peaks, one solution to identify all the peaks in the chromatogram is to use a deconvolution software like AMDIS prior to library searching [14].

### Data processing

After exported from MarkerLynx, All the collected data from the plasma samples were processed with SIMCA-P plus (version10.0, Umetrics AB, Sweden) as described previously [12,13], in which a range of multivariate statistical analysis were conducted. A data matrix was constructed with the sample IDs as observations and the peaks/retention times as the response variables. It was represented in a K-dimensional space (where K stands for the number of variables), and then projected and reduced to a few principal components that can maximize the separation between classes [15]. Here, partial least square discriminant analysis (PLS-DA) was used to process the required GC/TOFMS data. For PLS analysis, data were visualized by plotting principal component (PC) scores. In the scores, each point represents an individual sample. The plots allow the recognition of clusters of samples with similar scores. Each score plot has a loading plot associated with it, which allows in identifying the spectral regions (metabolites) responsible for the sample clustering observed [3].

To construct a regression model, multivariate regression analysis was performed based on 56 plasma samples at 5 h after GalN/LPS treatment using the Statistical Package for Social Scientists. The analysis was carried out with the binary response being dead or survived. This model was performed with a model group of 13 randomly selected survival samples and 13 dead samples. A validation group consisted of the remained 30 samples. The linear combination of those biomarkers identified in the metabonomic analysis was then applied to the logistic regression formula to predict the probability of FHF prognosis based on a formula score.

## Results

### Assessment of liver injury at 5 h and 6 h after treatment

Compared with plasma levels of ALT, AST, and TBIL in the survival group, they were significantly increased in the dead group at 6 h after GalN/LPS treatment. However, plasma ALT, AST, and TBIL levels showed no significant differences between the two groups at 5 h time point (Table 1).

**Table 1:** Assessment of liver injury at 5 h and 6 h after GalN/LPS treatment

Parameters		Survival group	Dead group	*P*
5 h	ALT (U/L)	698.52 ± 256.37	722.34 ± 242.87	4.13E-01
	AST (U/L)	712.45 ± 301.23	745.21 ± 297.38	5.42E-02
	TBIL (μmol/L)	3.12 ± 0.96	3.23 ± 1.13	1.75E-01
6 h	ALT (U/L)	762.12 ± 212.51	1967.34 ± 1032.78	2.15E-03
	AST (U/L)	801.92 ± 246.73	1874.54 ± 952.65	4.02E-04
	TBIL (μmol/L)	3.37 ± 1.02	5.12 ± 1.31	3.23E-03

### Absence of ribital in plasma samples of BALB/c mice

**Figure 1:**
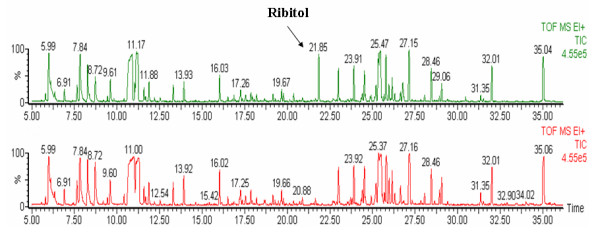
**Absence of ribital in plasma samples of BALB/c mice**. The peak of ribitol was showed in TIC of the sample with ribitol (upper), and there was no corresponding peak in the same retention time of TIC of another sample without ribitol (lower).

To confirm absence of ribitol in plasma of BALA/c mice, two samples from the same mouse were analyzed. Ribitol was added to one of them during plasma pretreatment. Figure 1 showed the peak of ribitol in the total ion current (TIC) of the sample with ribitol (upper), and there was no corresponding peak in the same retention time of TIC of another sample without ribitol (lower). Therefore, ribitol can be used as an internal standard to correct for minor variations during sample preparation and analysis.

### Partial least square-discriminant analysis

PLS is a supervised statistical method and additional knowledge (Y-variables) must be included to "supervise" the independent data (X-variables). In this study, discriminant analysis was performed using PLS. The PLS diagnostic is tabulated in Table 2 after modeling. Here R2X and R2Y are the fractions of X-variation and Y-variation modeled for specific component, respectively. Q2Y is the estimate of how well the model predicts the Y-variables through a default 7-fold cross-validation. The cumulative values of R2Y and Q2Y of the selected components close to one indicate a perfect fitting. So the diagnostic results indicated both the PLS models of 5 h and 6 h after GalN/LPS treatment demonstrated good performances based on the acquired metabonomics data.

**Table 2:** Modeling diagnostic of the metabolic data derived from plasma samples of various time points after GalN/LPS treatment

PLS model	Components	Modeling diagnostic		
		
		R2X	R2Y	Q2Y
4 h	1	0.186	0.288	0.225
	2	0.256	0.412	0.368
	3	0.335	0.556	0.406
5 h	1	0.351	0.742	0.701
	2	0.471	0.876	0.812
	3	0.504	0.915	0.857
6 h	1	0.367	0.781	0.666
	2	0.433	0.896	0.817
	3	0.493	0.941	0.862

Note. 1, 2, or 3 indicates quantities of components contributed to cumulative values. R2X is the fraction of X-variation; R2Y is the fraction of Y-variation; Q2Y is the cumulated cross-validation for R2Y. R2X, R2Y and Q2Y are all ranged from 0 to 1.

### Identification of metabolic markers for distinguishing between the survival and dead groups

**Figure 2:**
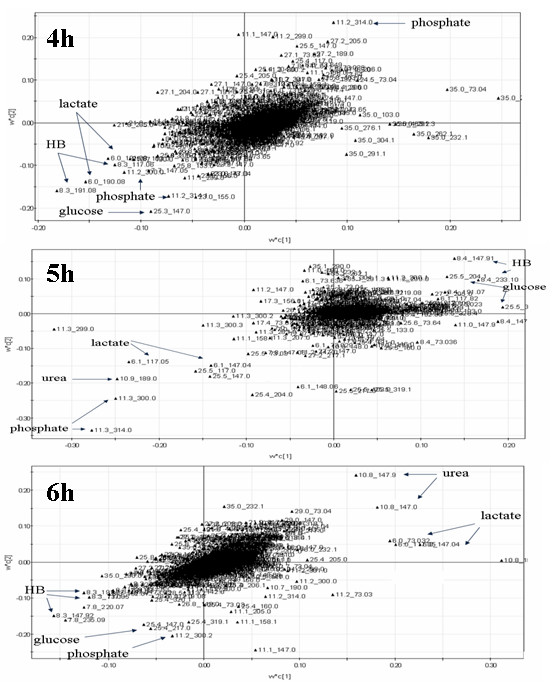
**Identification of metabolic markers for distinguishing between survival and dead groups**. Phosphate, HB, urea, glucose and lactate concentrations in plasma had the highest weightings on the clustering differences at 4 h, 5 h and 6 h after GalN/LPS treatment, although there was no clear cluster at 4 h.

In previous studies, clustering analysis of data collected at 4 h, 5 h and 6 h was performed using PLS-DA. Score plots showed distinct clustering differences between the survival and dead groups at 5 h and 6 h after GalN/LPS treatment. Loading plots reveal the impact of metabolites on the clustering results: the closer to zero, the smaller the influence of a linear metabolite combination. All of the three loading plots in Figure 2 suggested that phosphate, HB, urea, glucose and lactate concentrations in plasma had the highest weightings on the clustering differences although there was no clear cluster at 4 h.

### Regression analysis of data at 5 h point

Backward stepwise, which is one of three main approaches to automating the selection of explanatory variables for inclusion in a regression model, was used in the multivariate logistic regression analysis. In backward selection, all variables are included in the initial model, and then removed sequentially until a final model is produced. After 3 steps, lactate and glucose were removed, and other variables (HB, urea and phosphate) were used to calculate a nomogram. The derived nomogram, the death/survival index (DSI) was:

Probability of survival as compared to death was calibrated from the logistic regression analysis using the equation as follows:

The resulting value was defined as the DSI, with DSI < 0 incrementally favored a prognosis of "death", while DSI > 0 corresponded to a higher likelihood of a prognosis of "survival".

### Validation of the death/survival index (DSI)

**Figure 3:**
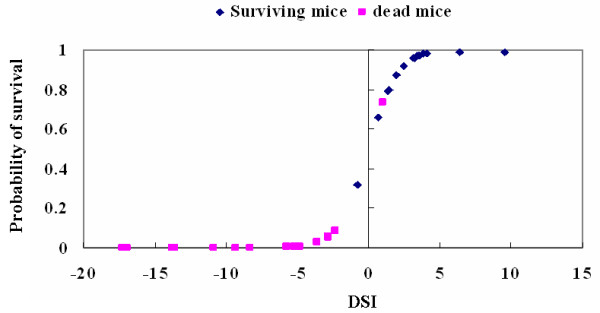
**Predicted probability of survival based on the death/survival index (DSI)**. DSI of >0.65 or < - 0.65 provided a relatively clear diagnosis, corresponding to a 93.3% or 6.7% probability of survival. Squares represent dead mice, and diamonds represent surviving mice.

We further validated and calibrated the DSI by comparing the predicted probability of FHF prognosis based on DSI with the validation group. Based on the cut-off value of DSI as above, one of 15 survival samples and one of 15 dead samples were removed, and general accurate rate of prediction was 93.3%. Figure 3 shows predicted probability of survival based on DSI. DSI of >0.65 or < - 0.65 provided a relatively clear diagnosis, corresponding to a 93.3% or 6.7% probability of survival.

## Discussion

It is well known that various biological factors affect the metabolic composition of biological fluids [15]. They are intrinsic (e.g. age, gender) and extrinsic (e.g. diet, smoking, drugs). External variation can be minimized in studies with laboratory animals, whereas in humans these factors are more difficult to control. Alternatively, because of higher fees, more explicit method and larger data, it is difficult to directly apply GC/TOFMS for clinical practice now. However, it is easy and applicable to construct a scoring system for early identification of diseases' prognosis based on metabolic biomarkers.

The rapidly developing field of metabonomics offered a new method to identify biomarkers. Nuclear magnetic resonance (NMR) and mass spectrometry (MS) are the most frequently employed methods of detection in the analysis of the metabolome [16,17]. NMR provides a rapid, non-destructive, high-throughput method that requires little or no sample preparation, and however, its equipment cost is much higher and its sensitivity is lower limited than MS. The most advantages of MS are its high sensitivity, and high-throughput. Furthermore, the combination of separation techniques with MS tremendously expands the capability of the chemical analysis of highly complex biological samples. Among the tandem techniques investigated, the coupling of capillary gas chromatography to MS (GC/MS) proved to be a potentially useful method based on its high sensitivity, peak resolution and reproducibility. The availability of GC/MS electron impact (EI) spectral libraries further facilitates the identification of diagnostic biomarkers and aids the subsequent mechanistic elucidation of the biological or pathological variations [14].

In the clinical arena, it is frequently important to identify the prognosis of FHF patients (survival or death) as this will influence patient management and candidacy for liver transplantation. Despite a number of novel and sometimes expensive biomarkers that have been developed and evaluated, most of these have not been reproducibly demonstrated to perform more effectively than those traditional and inexpensive laboratory values [4,8]. GC/TOFMS of biofluids and tissues coupled with appropriate statistical methods offer a novel and robust approach for identifying individual metabolites or combinations of metabolites that may serve as pathological biomarkers. This results in the generation of an endogenous metabolic profile that has the potential to reveal characteristics indicative of disease status.

To construct a model for early prediction of FHF prognosis, in previous studies we investigated the dynamics of metabolic intermediates and metabolic profiles using a GalN/LPS-treated BALB/c mouse model of FHF prior to clinical FHF patients [12,13]. In this study, PLS-DA was used to obtain a list of potential biomarkers which are statistically significant and which separate one class from another. The PLS diagnostic tabulated in Table 2 contained three factors, showing the performance statistics of R2X, R2Y and Q2Y. R2X is the cumulative modeled variation in the GC/TOFMS response variables, X; R2Y is the cumulative modeled variation in the observation variables, Y; and Q2Y is the cumulative predicted variation in Y, according to cross-validation. The ranges of these parameters are 0-100%, where 100% indicates a perfect fit [18]. So the diagnostic results indicated both the PLS models of 5 h and 6 h after GalN/LPS treatment demonstrated good performances based on the acquired metabonomics data. That means distinct clustering (survival and death) was obtained at 5 h and 6 h time points. To construct an early prediction model, we selected 5 h time point when there was no significant difference in plasma levels of ALT, AST, and TBIL between the two groups. Loadings plot represented the impact of metabolites on the clustering results: the closer to zero, the smaller the influence of a linear metabolite combination. Phosphate, HB, urea, glucose and lactate concentrations in plasma had the highest weightings on the clustering differences at the three time points, although there was no clear cluster at 4 h after treatment. It was suggested that a combination of phosphate, HB, urea, glucose and lactate concentrations in the plasma could potentially be used to separate the survival and dead groups earlier.

Based on the set of potential metabolic biomarkers including phosphate, HB, urea, glucose and lactate identified using GC/TOFMS combined with multivariate statistics, we attempted to form a scoring system to early identify FHF outcomes. By the multivariate logistic regression analysis, lactate and glucose were removed, and then the death/survival index (DSI) was constructed based on relative concentrations of other variables (HB, urea and phosphate) and validated well in the samples from independent validation group. The DSI maintains important and unique methodological aspects that enhance its utility as compared to other studies designed to predict FHF prognosis. The use of logistic regression facilitated appropriate weighting of the parameters that comprise the DSI. Alternatively, the DSI is a continuous variable, we can identify the probability of FHF prognosis (survival or death) based on the magnitude and dynamic change of the DSI.

## Conclusions

We have developed a novel scoring system that is highly accurate in predicting the prognosis of FHF mouse model. The methodological aspects based on metabonomic technologies combining with multivariate logistic regression were constructed and may be referred in clinical practice. As a useful prognostic tool, it may be complemented with other available information such as traditional laboratory values and clinical parameters.

## Potential limitations

Various biological factors affect the metabolic composition of biological fluids, and these factors might be involved in humans to different extend. Although our previous pilot result showed that changes of plasma phosphate, HB, urea, glucose and lactate levels in FHF patients were consistent with those of FHF mouse model, it is unknown whether the scoring system based on the five metabolites would be applicable to predicting the prognosis FHF seen in clinical practice. In addition, etiology of FHF patients is complex, which can influence metabonomics of body fluids. All of these need a further investigation.

## Abbreviations

FHF: fulminant hepatic failure; GalN: d-galactosamine; LPS: lipopolysaccharide; HB: β-hydroxybutyrate; GC/TOFMS: gas chromatography/time-of-flight mass spectrometry; PLS-DA: partial least square discriminant analysis; TIC: total ion current; DSI: death/survival index

## Competing interests

The authors declare that they have no competing interests.

## Authors' contributions

BF contributed to design, experimental process, data acquisition, statistical analysis and drafted the manuscript. SMW contributed to experimental process, data acquisition, statistical analysis and drafted the manuscript. SL participated in experimental process and analysis. FL participated in study planning, data acquisition and statistical analysis. HSC contributed to data analysis and to the manuscript. YG participated to planning and statistical analysis. FTD contributed to study design, data analysis and drafting of the manuscript. LW participated in design, funding analysis and manuscript drafting. All authors read and approved the final manuscript.

## Pre-publication history

The pre-publication history for this paper can be accessed here:

http://www.biomedcentral.com/1471-230X/9/99/prepub
